# Molecular Epidemiology, Genetic Diversity, and Isolation of Porcine Reproductive and Respiratory Syndrome Virus in Hubei Province, Central China, 2024–2025

**DOI:** 10.3390/ani16213367

**Published:** 2026-10-25

**Authors:** Chang Li, Keli Yang, Hao Liu, Wei Liu, Qiong Wu, Ting Gao, Jiajia Zhu, Fangyan Yuan, Rui Guo, Danna Zhou

**Affiliations:** 1 Key Laboratory of Prevention and Control Agents for Animal Bacteriosis, Ministry of Agriculture and Rural Affairs, Key Laboratory of Animal Pathogenic Microbiology of Hubei Province, Institute of Animal Sciences and Veterinary Medicine, Hubei Academy of Agricultural Sciences, Wuhan 430064, China; lichang1113@hbaas.ac.cn (C.L.); keliy6@hbaas.ac.cn (K.Y.); 17509677632@163.com (H.L.); liuwei@hbaas.ac.cn (W.L.); wuqiong302@hbaas.ac.cn (Q.W.); gaoting2017@hbaas.ac.cn (T.G.); xmszjj@hbaas.ac.cn (J.Z.); fyyuan@hbaas.ac.cn (F.Y.); guorui@hbaas.ac.cn (R.G.); 2 College of Veterinary Medicine, Anhui Agricultural University, Hefei 230036, China

**Keywords:** PRRSV, Hubei province, NADC30-like, NADC34-like, genetic recombination, virus isolation, selection pressure

## Abstract

Porcine reproductive and respiratory syndrome virus (PRRSV) remains the most economically devastating pathogen of the global swine industry, with frequent genetic recombination and continuous lineage replacement driving its rapid evolution. Hubei Province, a major swine-producing hub in central China, endures substantial PRRSV disease pressure, yet the current epidemiological and evolutionary landscape of circulating strains remains poorly characterized. In this study, 1478 clinical samples were collected from 12 prefecture-level cities between 2024 and 2025 and subjected to RT-PCR detection, ORF5 sequencing, virus isolation, and complete genome analysis. The detection rate among clinical samples was 26.32%, highest in nursery pigs (32.65%). Phylogenetic analysis of 136 ORF5 sequences revealed lineage 1 dominance (66.18%), comprising NADC30-like (38.24%) and NADC34-like (27.94%) strains, followed by lineage 8 (24.26%), lineage 5 (5.15%), and lineage 3 (4.41%). Six isolates were recovered, and whole-genome analysis identified lineage-specific NSP2 deletion patterns and a recombinant strain (LW HP) bearing a JXA1-like backbone with NADC30-like insertions. Selection pressure analysis ranked Nsp2 as the least-constrained gene (dN/dS = 0.3065; purifying selection), with positively selected codons in GP5 and the NSP2 hypervariable region. These findings delineate the current epidemic status and genetic characteristics of PRRSV in central China, providing critical data for regional prevention and vaccine development.

## Simple Summary

Porcine reproductive and respiratory syndrome is one of the most devastating viral diseases affecting pigs worldwide, causing reproductive failure in sows and severe respiratory disease in growing pigs, and inflicting enormous economic losses on the swine industry. The causative virus evolves rapidly and frequently exchanges genetic material between strains, making disease control increasingly difficult. Hubei Province, a major pig-producing region in central China, has long suffered from this disease, but the current epidemic situation remains poorly understood. In this study, we examined nearly 1500 clinical samples collected from pig farms across 12 cities in Hubei between 2024 and 2025. We found that about one in four of the samples tested positive, with nursery pigs showing the highest infection rate. Genetic analysis revealed that recently emerged North American-type strains have become dominant, replacing older classical strains. Six live viruses were successfully isolated and their complete genomes were sequenced, uncovering a novel recombinant strain and distinct genetic patterns across virus types. These findings provide up-to-date information on disease circulation in central China, which will help guide local vaccination strategies, strengthen disease surveillance, and support the development of more effective vaccines.

## 1. Introduction

Porcine reproductive and respiratory syndrome (PRRS), caused by PRRS virus (PRRSV), is a globally endemic disease characterized by reproductive failure in sows and respiratory distress in growing pigs, imposing enormous economic losses on the swine industry worldwide [1,2,3]. As an enveloped, single-stranded positive-sense RNA virus of the family Arteriviridae, PRRSV displays an exceptionally high mutation rate and undergoes frequent inter-lineage recombination [4,5], both of which accelerate viral evolution and hamper disease prevention and control [6,7]. Based on genomic divergence, PRRSV is classified into two distinct genotypes: PRRSV-1 (European type) and PRRSV-2 (North American type), sharing only approximately 60% nucleotide identity [8,9]. In China, PRRSV-2 has been the dominant genotype since its first recognition in 1996, with the epidemiological landscape undergoing successive lineage shifts over the past three decades [10,11].

Historically, highly pathogenic PRRSV (HP-PRRSV, sublineage 8.7, sublineage L8E), represented by the JXA1 strain, emerged in 2006 and rapidly became the predominant strain in China, causing severe morbidity and mortality in pigs of all ages [12,13,14]. Since its initial detection in 2013, NADC30-like PRRSV (sublineage 1.8, sublineage L1C) has spread rapidly across the country, driven by its high recombination propensity and capacity to evade vaccine-induced immunity [15,16,17,18,19]. More recently, NADC34-like PRRSV (sublineage 1.5, sublineage L1A), another lineage 1 variant, has been identified in an increasing number of provinces and is gradually becoming endemic, further complicating the national epidemiological landscape [20,21,22,23,24,25]. Large-scale molecular epidemiological surveys have confirmed that lineage 1 strains (NADC30-like and NADC34-like) have gradually displaced sublineage 8.7 as the dominant PRRSV populations in China, while frequent recombination between lineage 1 and other lineages has generated novel recombinant strains with altered pathogenicity and tissue tropism [16,26,27,28].

Reliable broad-spectrum nucleic acid detection is fundamental to PRRSV epidemiological surveillance. The nsp7 gene and 3′ untranslated region (3′UTR) are highly conserved across PRRSV-2 lineages and are widely employed as targets for universal RT-PCR assays, thereby minimizing false negatives arising from sequence variation in hypervariable regions [13,29]. The ORF5 gene, encoding the major envelope glycoprotein GP5, serves as the standard marker for PRRSV lineage classification and phylogenetic tracing. In parallel, the NSP2 coding region exhibits lineage-specific amino acid deletions and represents a hotspot for inter-lineage recombination, both of which are further resolved by complete genome sequencing [30,31,32]. This tiered strategy comprises conserved-region screening, ORF5 population sequencing, and representative complete-genome analysis, thereby ensuring detection reliability while comprehensively delineating the molecular epidemiological characteristics of circulating strains.

Hubei Province, located in central China, is a major hub for swine production and pig movement. The region hosts a high density of intensive farms and experiences frequent cross-regional transport of breeding stock and market pigs, thereby elevating the risk of PRRSV introduction and dissemination, which is further compounded by the interplay between viral evolution and host immune pressure [3,33]. Earlier regional surveillance indicated that HP-PRRSV previously predominated in Hubei, with vaccine-related strains commonly detected in immunized herds [3,34,35]. However, following the nationwide expansion of lineage 1 PRRSV, the contemporary lineage composition, selection pressure profiles, and recombination dynamics in Hubei remain poorly defined, and locally isolated and fully characterized epidemic strains are lacking.

To address these unresolved questions, a systematic etiological investigation of PRRSV was conducted across 12 prefecture-level cities in Hubei Province between 2024 and 2025. The workflow comprised universal RT-PCR targeting the conserved ORF7/3′UTR region for primary screening; ORF5 sequencing for lineage classification and population-level genetic variation analysis; single-virus whole-genome sequencing of representative isolates (Illumina NovaSeq PE150) for recombination breakpoint identification; virus isolation in primary porcine alveolar macrophages (PAMs), with subsequent adaptation to MARC-145 cells [36,37,38,39]; and ultrastructural characterization of adapted isolates by transmission electron microscopy. This study provides updated molecular epidemiological data for PRRSV in central China and offers well-characterized local isolates to support the development of region-specific prevention and control strategies.

## 2. Materials and Methods

### 2.1. Sample Collection and Processing

Between January 2024 and December 2025, 1478 clinical samples were collected from pig farms across 12 prefecture-level cities of Hubei Province. The panel comprised oral fluid, testicular fluid, serum, lung tissue, and lymph nodes from pigs exhibiting fever, cough, dyspnea, abortion, or stillbirth. Tissue specimens (~0.5 g) were homogenized in 2 mL sterile PBS and centrifuged at 12,000 rpm for 10 min at 4 °C; supernatants were aliquoted and stored at −80 °C. All procedures were approved by the Animal Care and Use Committee of Hubei Academy of Agricultural Sciences (approval no. 2025SC022).

### 2.2. RNA Extraction and RT-PCR Detection of PRRSV-2

Total RNA was extracted from 200 μL of fluid or 10% tissue homogenate using AIDzol Reagent (Aidlab, Beijing, China) per the manufacturer’s protocol. Primers targeting the ORF7/3′UTR of PRRSV-2 (P1: 5′-ATGGCCAGCCAGTCAATCA-3′; P2: 5′-TCGCCCTAATTGAATAGGTGACT-3′) were used for viral detection. RT-PCR was performed with the HiScript II One Step RT-PCR Kit (Vazyme, Nanjing, China) in a 25 μL reaction containing 12.5 μL 2 × One Step Mix, 1 μL each primer (10 μmol/L), 1 μL Enzyme Mix, 3 μL RNA template, and 6.5 μL RNase-free water. Thermal cycling consisted of 50 °C for 30 min, 94 °C for 5 min, 35 cycles of 94 °C for 30 s, 55 °C for 30 s, and 72 °C for 30 s, followed by a final extension at 72 °C for 5 min. Amplicons were resolved on 1% agarose gels stained with GoldView and visualized under UV light. Samples yielding a 433 bp product were considered PRRSV-positive.

### 2.3. Amplification and Sequencing of the ORF5 Gene

PRRSV-positive samples were further analysed by full-length ORF5 amplification. Primers flanking the ORF5 gene (ORF5-F: 5′-GGGCAACCGTTAGCCTGTC-3′; ORF5-R: 5′-AAACGCCAAAAAGCCCTTCTG-3′) were designed against conserved regions of PRRSV-2 reference strains [40]. RT-PCR conditions were: 50 °C for 30 min, 94 °C for 5 min, 35 cycles of 94 °C for 30 s, 56 °C for 30 s, and 72 °C for 1 min, followed by 72 °C for 5 min. Amplicons with distinct bands were gel-purified (Omega Bio-tek, Norcross, GA, USA) and directly Sanger-sequenced (Qingke Biotechnology, Beijing, China).

### 2.4. Complete Genome Sequencing and Assembly

Whole-genome sequencing was performed on six PRRSV isolates recovered from PRRSV-positive clinical samples. Isolates were included for sequencing if they yielded a complete viral genome with sufficient sequencing coverage, with no additional selection based on lineage or geographic origin. Viral RNA was extracted from MARC-145-adapted cultures at the fifth passage (P5) using AIDzol Reagent (Aidlab, Beijing, China) and assessed by agarose gel electrophoresis and Qubit fluorometry. Single-virus whole-genome sequencing was performed by Magigene Co., Ltd. (Guangzhou, China) on the Illumina NovaSeq 6000 platform (PE150, ~1 Gb raw data per sample). Raw reads were trimmed with Trimmomatic v0.39, depleted of host (Sus scrofa) and rRNA sequences, and assembled against PRRSV-2 reference genomes, followed by contig scaffolding and variant calling (SNP/InDel). Consensus sequences were validated by remapping clean reads to ensure coverage accuracy. Genome annotation was based on the canonical PRRSV-2 organization (5′UTR–ORF1a/ORF1b–ORF2a/2b–ORF3–ORF4–ORF5–ORF6–ORF7–3′UTR).

### 2.5. Phylogenetic and Sequence Variation Analysis

ORF5 and whole-genome sequences were aligned with lineage-representative strains retrieved from GenBank using MAFFT v7.0 [41]. Phylogenetic trees were constructed by Maximum Likelihood using IQ-TREE 2.4.0, with the best-fit nucleotide substitution model (TVM + F + I + R3 for ORF5) selected by ModelFinder (IQ-TREE 2.4.0) based on the Bayesian Information Criterion. Node support was evaluated with 1000 ultrafast bootstrap replicates [42,43,44]. GP5 and NSP2 amino acid sequences were aligned in parallel, and key functional regions—signal peptide, neutralizing epitope, decoy epitope, and lineage-specific deletion regions—were interrogated. Pairwise nucleotide and amino acid identity matrices were calculated in MEGA 12 [45]. GP5 substitutions and deletions were mapped against the prototype PRRSV-2 strain VR-2332. Lineage and sublineage designations follow the numerical system of Shi et al. (2010), with cross-reference to the refined classification of Yim-im et al. (2023): sublineage 1.5 = L1A, sublineage 1.8 = L1C, lineage 3 = L3, lineage 5 = L5, and sublineage 8.7 = L8E [18,19].

### 2.6. Recombination Analysis

Whole-genome sequences of the six isolates were screened for recombination using RDP4 v4.10 with seven embedded methods (RDP, GENECONV, BootScan, MaxChi, Chimaera, SiScan, and 3Seq). Events supported by ≥4 methods at *p* < 10^−6^ were considered significant [46]. Breakpoints and the reference strain showing the closest similarity in each genomic segment, were further examined by SimPlot v3.5.1 with a 200 bp sliding window and 20 bp step size.

### 2.7. Virus Isolation and Identification

Primary PAMs were obtained from 4-week-old PRRSV-negative piglets by bronchoalveolar lavage and stored in liquid nitrogen. PRRSV-positive samples were filtered through 0.22-μm membranes and inoculated onto PAMs (RPMI 1640, 10% FBS). After 2 h adsorption at 37 °C with 5% CO_2_, the inoculum was replaced with fresh medium. Cytopathic effects (CPE) were monitored daily; upon appearance of overt CPE, cultures were freeze-thawed three times and supernatants harvested for serial passage on PAMs (P1–P2). At P2, supernatants were transferred to MARC-145 cells (DMEM, 10% FBS) for further adaptation and passage (P3–P5). Fifth-passage (P5) stocks were confirmed by ORF7/3′UTR RT-PCR and IFA using a PRRSV N-specific polyclonal antibody (GeneTex, Irvine, CA, USA) [36,47] and used for whole-genome sequencing. Eighth-passage (P8) stocks were used for virus titration and biological characterization. The virus titer (50% tissue culture infectious dose, TCID_50_) was calculated by the Reed–Muench method [48].

### 2.8. Growth Kinetics

MARC-145 cells were seeded in 24-well plates and grown to confluence (~1.5 × 10^5^ cells per well). Monolayers were inoculated with each isolate at a multiplicity of infection (MOI) of 0.1 in maintenance medium (DMEM containing 2% FBS and 1% penicillin–streptomycin). After 2 h of adsorption at 37 °C with 5% CO_2_, the inoculum was removed and replaced with fresh maintenance medium. At 6, 12, 18, 24, 36, 48, 60, 72 and 96 h post-infection, the supernatant and cells from three wells were collected separately, subjected to three freeze–thaw cycles and stored at −80 °C. Viral titers in each sample were determined by TCID_50_ assay on MARC-145 cells and expressed as log_10_TCID_50_/mL. Growth curves were plotted as the mean ± SD of the three wells at each time point (*n* = 3).

### 2.9. Transmission Electron Microscopy

MARC-145 cells were inoculated with PRRSV isolates (P8) at an MOI of 0.1. At ~70% CPE, cells were harvested by trypsinization and pelleted at 1000× *g* for 10 min. Pellets were fixed in 2.5% glutaraldehyde (0.1 M phosphate buffer, pH 7.4) at 4 °C overnight, rinsed, and post-fixed in 1% osmium tetroxide for 2 h at room temperature. After dehydration through a graded ethanol series, samples were permeabilized with propylene oxide, embedded in Epon 812, and polymerized at 60 °C for 48 h. Ultrathin sections (60–80 nm) were stained with uranyl acetate and lead citrate and examined under a Hitachi H-7650 TEM (Hitachi, Tokyo, Japan) at 80 kV.

### 2.10. Selection Pressure Analysis

Positively selected codons in the 136 ORF5 sequences and the NSP2 hypervariable region were identified using MAFFT-aligned codon-based alignments. Analyses were performed in HyPhy v2.5.50 implemented on the Datamonkey 2.0 web server using four methods: SLAC, FEL, MEME, and FUBAR [49]. Significance was set at *p* < 0.1 for SLAC, FEL, and MEME, and posterior probability > 0.9 for FUBAR. Site-wise dN/dS ratios were additionally computed by the Nei–Gojobori method with Jukes–Cantor correction. Sites supported by ≥3 methods were considered robustly selected. Gene-wise dN/dS ratios were calculated in MEGA 12 using the Nei–Gojobori method [50].

### 2.11. Amino Acid Variation and Diversity Landscape

Amino acid variation rates were computed for each GP5 functional domain within each of the five PRRSV lineages identified. The variation rate was defined as the percentage of positions bearing ≥ 2 distinct residues among strains of the same lineage. Eleven domains were assessed: signal peptide (aa 1–26), decoy epitope A (aa 27–30), HVR1 (aa 32–36), putative neutralizing epitope B (PNE; aa 37–45), HVR2 (aa 57–61), TM1 (aa 66–93), TM2 (aa 99–105), TM3 (aa 111–127), T-cell epitope 1 (aa 119–128), T-cell epitope 2 (aa 151–159), and B-cell epitope (aa 179–200). Site-wise amino acid diversity was quantified by Shannon entropy across the 136 field sequences (range: 0 = completely conserved; ~4.32 = all 20 residues equally frequent).

### 2.12. N-Linked Glycosylation Site Prediction

Potential N-linked glycosylation sites were predicted by scanning all 136 GP5 sequences for the canonical N-X-S/T motif (X ≠ P). The frequency of each intact motif among the 136 field strains was calculated, alongside its presence or absence in the five reference strains (NADC34, NADC30, QYYZ, VR-2332, JXA1).

### 2.13. Statistical Analysis

Positive rates and lineage proportions were presented descriptively. Ninety-five percent confidence intervals for proportions were calculated using the Wilson method. Between-city heterogeneity in detection rate was assessed with a χ^2^ test of homogeneity on a 2 × 12 contingency table, and each city was compared with the remaining cities pooled using Fisher’s exact test with Benjamini–Hochberg control of the false discovery rate. The difference in detection rate between 2024 and 2025 was assessed with a two-proportion z-test (equivalent to Pearson’s χ^2^ test of association without continuity correction). Analyses were performed in GraphPad Prism v10.1.0 and R v4.3.1; data are expressed as means ± SD.

## 3. Results

### 3.1. PRRSV Detection in Clinical Samples

**Figure 1:**
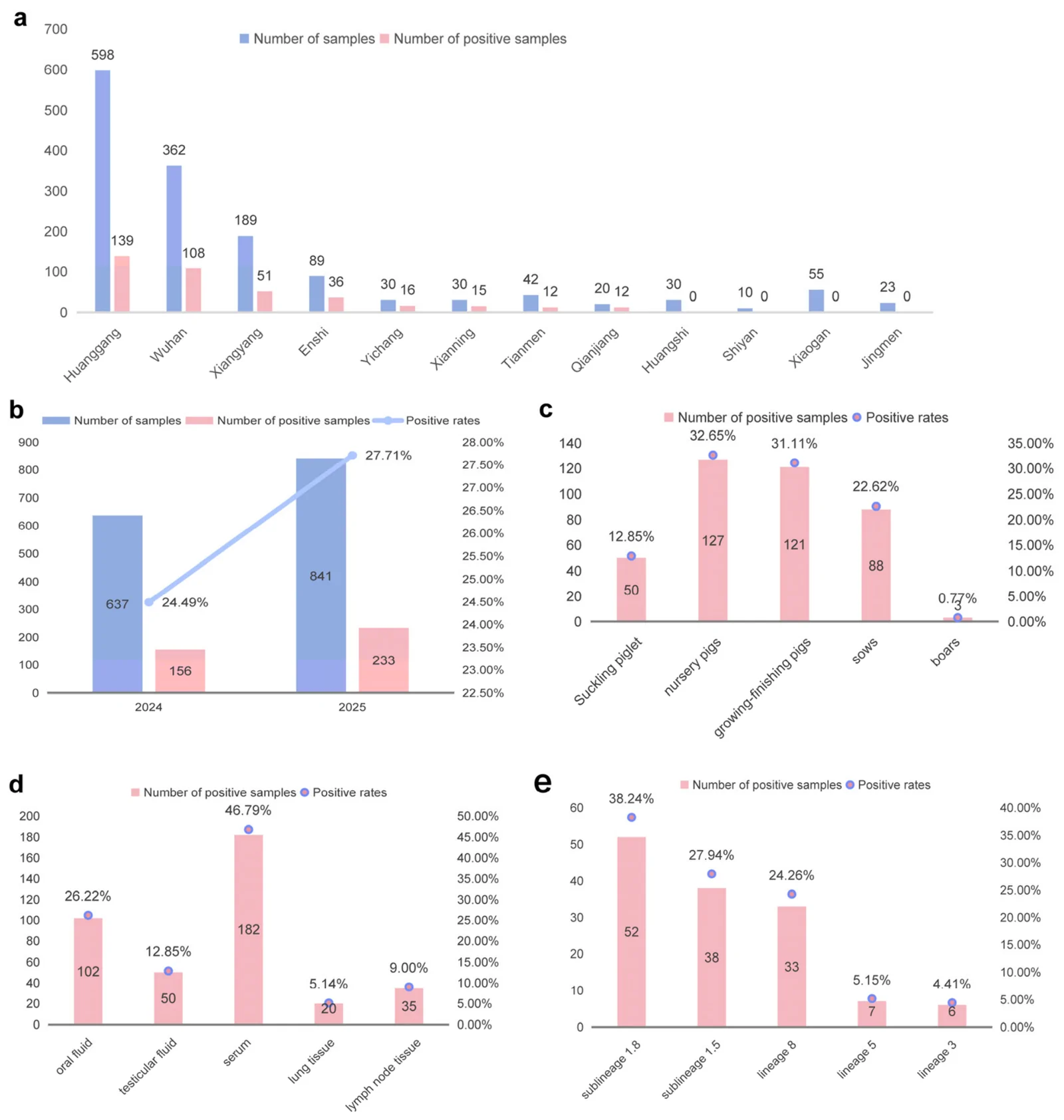
Epidemiological surveillance of PRRSV in Hubei Province, 2024–2025. (**a**) Geographic distribution of samples (*n* = 1478) and PRRSV-positive cases (*n* = 389) across 12 prefecture-level cities. (**b**) Annual detection rate, 24.49% in 2024 and 27.71% in 2025 (difference not statistically significant, *p* = 0.165). (**c**) Age-group distribution of positive samples, with nursery pigs predominating (32.65%). (**d**) Sample-type distribution, including oral fluid, testicular fluid, serum, lung tissue, and lymph node. (**e**) Lineage composition of 136 ORF5 sequences, with lineage 1 (NADC30-like and NADC34-like) predominating at 66.18%.

Of 1478 samples tested by ORF7/3′UTR RT-PCR, 389 were PRRSV-positive (26.32%; Table 1). A numerically higher detection rate was observed in 2025 (27.71%, 233/841) than in 2024 (24.49%, 156/637) (Figure 1b); this difference was not statistically significant (two-proportion z-test, z = 1.39, *p* = 0.165; risk difference +3.22 percentage points, 95% CI −1.29 to +7.72). Detection rates differed among the 12 cities (χ^2^ = 88.35, df = 11, *p* < 0.001). The numerically highest rates were recorded in Qianjiang (60.00%, 12/20), Yichang (53.33%, 16/30), Xianning (50.00%, 15/30) and Enshi (40.45%, 36/89), whereas Huangshi (0/30), Shiyan (0/10), Xiaogan (0/55) and Jingmen (0/23) yielded no positive samples (Table 1; Figure 1a). The number of samples per city was, however, highly unequal and sampling followed clinical submission rather than a random or proportional design. These city-level values therefore represent detection rates among submitted samples, not regional prevalence, and the wide 95% confidence intervals of the low-n cities (e.g., Shiyan, 0–27.8%; Qianjiang, 38.7–78.1%) preclude firm conclusions about true geographic differences. By age group, nursery pigs predominated (32.65%), followed by growing-finishing pigs (31.11%), sows (22.62%), suckling piglets (12.85%), and boars (0.77%) (Figure 1c). Serum yielded the highest positivity (46.79%), followed by oral fluid (26.22%), testicular fluid (12.85%), lymph node (8.99%), and lung tissue (5.14%) (Figure 1d) (Supplementary Table S1).

### 3.2. Phylogenetic Analysis of ORF5 Sequences

All 136 high-quality ORF5 sequences (Supplementary Table S2) obtained from positive samples belonged to PRRSV-2 and clustered within five lineages. Lineage 1 predominated (66.18%), comprising 52 sublineage 1.8 strains (NADC30-like, L1C, 38.24%) and 38 sublineage 1.5 strains (NADC34-like, L1A, 27.94%). The remaining strains fell within lineage 8 (HP-PRRSV-like, L8, 33 strains, 24.26%), lineage 5 (VR-2332-like, L5, 7 strains, 5.15%), and lineage 3 (QYYZ-like, L3, 6 strains, 4.41%) (Figure 1e). Lineage composition was broadly stable across years: in 2024 (*n* = 58), sublineages 1.8 and 1.5 accounted for 37.93% and 27.59%, lineage 8 for 25.86%, lineage 5 for 5.17%, and lineage 3 for 3.45%; corresponding figures for 2025 (*n* = 78) were 38.46%, 28.21%, 23.08%, 5.13%, and 5.13%, respectively (Supplementary Table S1).

**Figure 2:**
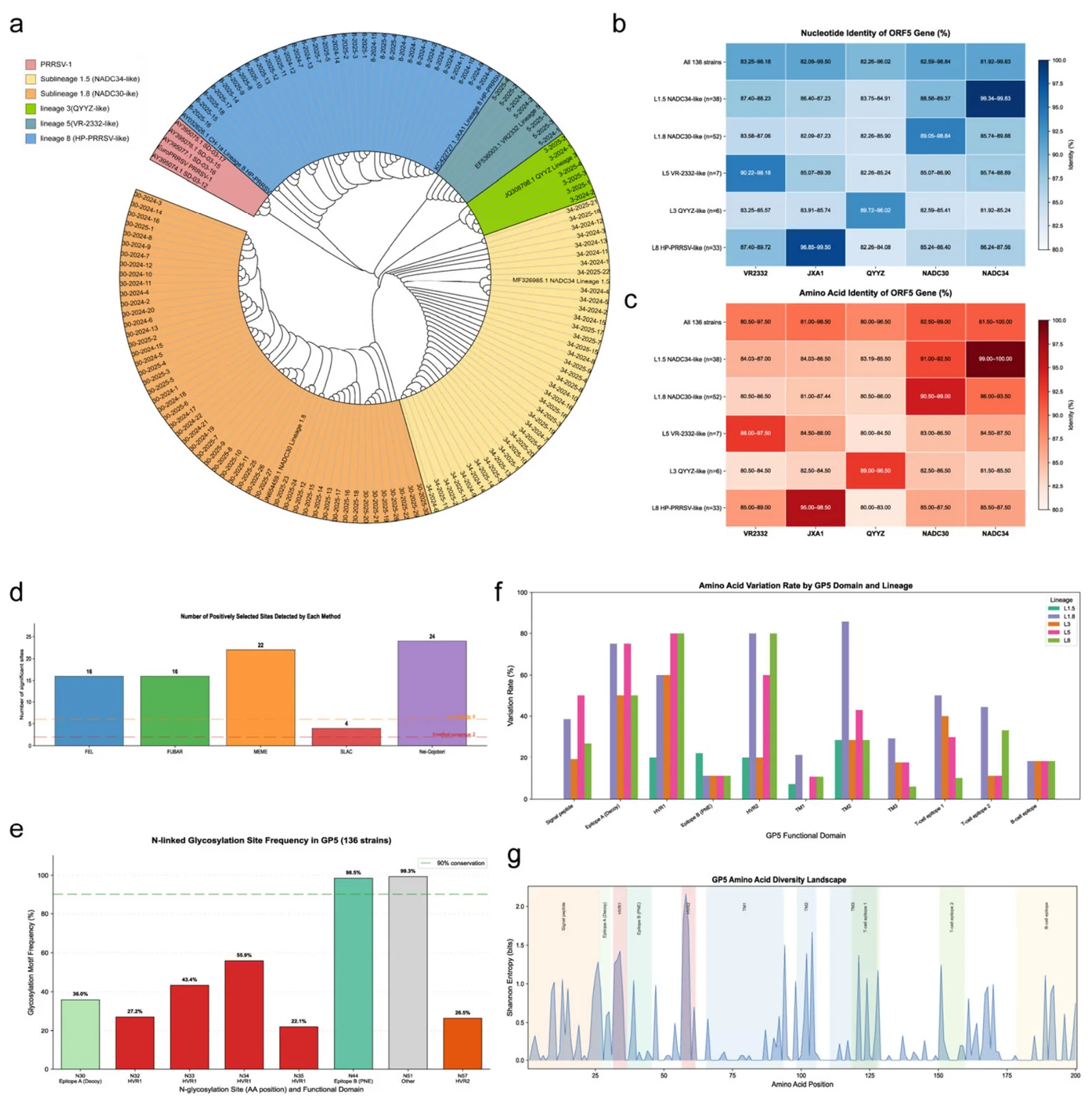
Phylogenetic and sequence variation analysis of ORF5. (**a**) Maximum Likelihood phylogenetic tree of ORF5 nucleotide sequences, showing distribution across five lineages. (**b**) Nucleotide identities of 136 ORF5 sequences relative to five reference strains (VR-2332, JXA1, QYYZ, NADC30, NADC34). (**c**) Amino acid identities relative to reference strains. (**d**) Positively selected sites in GP5 identified by five methods (FEL, FUBAR, MEME, SLAC, Nei–Gojobori); aa 32 and aa 59 were detected by all five. (**e**) Distribution of predicted N-linked glycosylation sites, with conserved residues at N44 and N51. (**f**) Amino acid variation in key antigenic regions (decoy epitope, PNE, T- and B-cell epitopes) across lineages. (**g**) Amino acid diversity landscape of GP5 (Shannon entropy).

Pairwise comparisons revealed that the 136 ORF5 sequences shared 83.3–98.2%, 82.3–99.5%, 82.3–96.0%, 82.6–98.8%, and 81.9–99.8% nucleotide identity with VR-2332, JXA1, QYYZ, NADC30, and NADC34, respectively. The 38 sublineage 1.5 (L1A) strains shared 99.3–99.8% identity with NADC34, whereas the 52 sublineage 1.8 (L1C) strains shared 89.1–98.8% identity with NADC30. Lineage 8 (L8) strains showed 96.8–99.5% identity with JXA1, lineage 5 (L5) strains shared 90.2–98.2% with VR-2332, and lineage 3 (L3) strains exhibited 89.7–96.0% identity with QYYZ (Figure 2b). Cross-lineage identities ranged from 82.1–89.9%, confirming clear genetic divergence. Amino acid identities mirrored nucleotide patterns (Figure 2c), supporting the lineage assignments (Figure 2a). Full identity matrices are provided in Supplementary Table S2.

Selection pressure analysis using five methods (FEL, FUBAR, MEME, SLAC, and Nei–Gojobori) revealed pervasive positive selection across GP5 (Figure 2d). Sixteen sites were supported by ≥3 methods, of which two (aa 32 and aa 59) were concordantly detected by all five, and six (aa 14, 32, 33, 34, 59, 102) by ≥4. Most robustly selected sites localized to the signal peptide (aa 1–26) and HVR1 (aa 32–36), with aa 32–34 yielding dN/dS ratios of 5.94–54.36. Site aa 102 in the ectodomain showed the highest dN/dS (24.04 by FUBAR), indicative of strong immune-driven selection. By contrast, the primary neutralizing epitope (PNE, aa 37–45) and transmembrane domains were largely conserved, with only aa 34 at the HVR1–PNE boundary under significant selection (Supplementary Table S3). Two predicted N-glycosylation sites were present in almost all field strains—N51 in 135/136 (99.3%) and N44 in 134/136 (98.5%), whereas N30, N32–35, and N57 varied by lineage; N34 occurred in 55.9% of strains (Figure 2e). Domain-wise variation was highest in HVR1 for lineage 5 and lineage 8 (both 80%), and in TM2 for sublineage 1.8 (86%); PNE and B-cell epitope remained conserved across lineages. Lineage-specific glycan patterns in decoy epitope A and HVR1, particularly at positively selected aa 34, suggest differential glycan shielding as a potential immune-evasion mechanism (Figure 2f). Shannon entropy peaked in HVR2 (aa 57–61, >2.0 bits), followed by HVR1 (aa 32–36, ~1.4 bits), decoy epitope (aa 27–30), and signal peptide; PNE (aa 37–45) and B-cell epitope (aa 179–200) showed low entropy (Figure 2g; Supplementary Table S4). The entropy landscape corroborated the dN/dS results, identifying decoy epitope A, HVR1, and HVR2 as the principal determinants of GP5 antigenic variation.

### 3.3. Virus Isolation and Biological Characteristics

**Figure 3:**
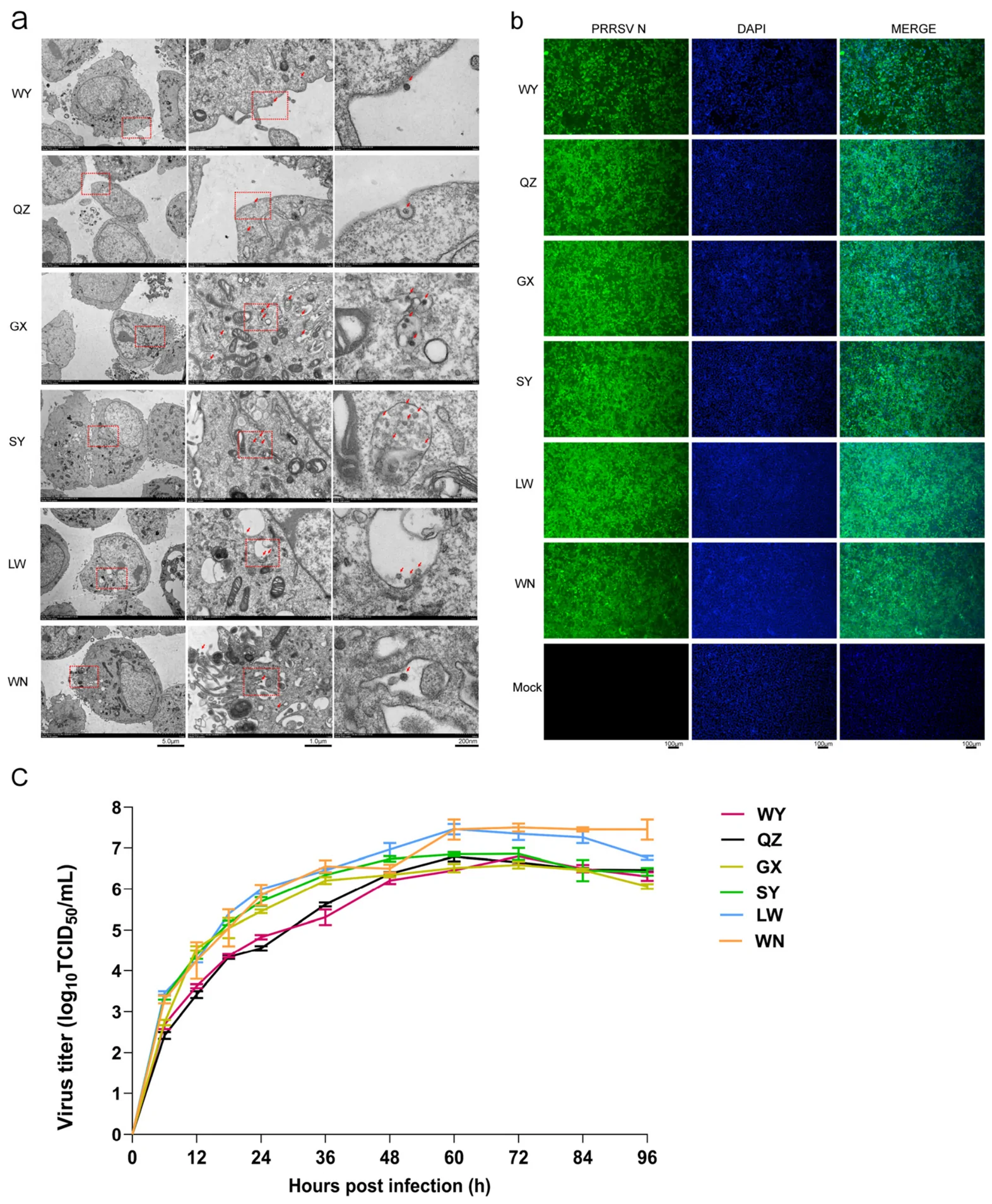
Biological characterization of isolated PRRSV strains. (**a**) TEM of ultrathin sections of PRRSV-infected MARC-145 cells, showing spherical enveloped virions (50–60 nm); red dashed boxes indicate the regions shown at higher magnification in the adjacent panel, and red arrows indicate virions. (**b**) IFA detection of PRRSV N protein in infected cells. (**c**) Growth kinetics of PRRSV isolates in MARC-145 cells at an MOI of 0.1. Error bars represent the standard deviation of three independent biological replicates (*n* = 3).

Six PRRSV strains (WY 30, QZ 30, GX 34, SY HP, LW HP, and WN HP) were isolated in primary PAMs and subsequently adapted to MARC-145 cells, producing typical CPE, namely, cell rounding, aggregation, and detachment, within 48–72 h. TEM of ultrathin sections revealed spherical enveloped virions (50–60 nm) with electron-dense nucleocapsid cores, predominantly in the cytoplasm, ER-derived vesicles, and perinuclear region, consistent with Arteriviridae morphology. Abundant double-membrane vesicles (DMVs), hallmark of arterivirus replication complexes, were distributed throughout the cytoplasm. Conspicuous cytopathology accompanied infection, including mitochondrial swelling with disrupted cristae, rough ER dilation, chromatin margination, and occasional pyknosis (Figure 3a). N-protein-specific IFA yielded abundant cytoplasmic fluorescence in infected MARC-145 cells (Figure 3b). Titers of eighth-passage stocks ranged from 10^5.8^ to 10^7.7^ TCID_50_/mL. In growth-kinetics assays (MOI 0.1), titers peaked at 60 h post-infection and declined slightly thereafter (Figure 3c).

### 3.4. Genomic Characterization of Six PRRSV Isolates

**Figure 4:**
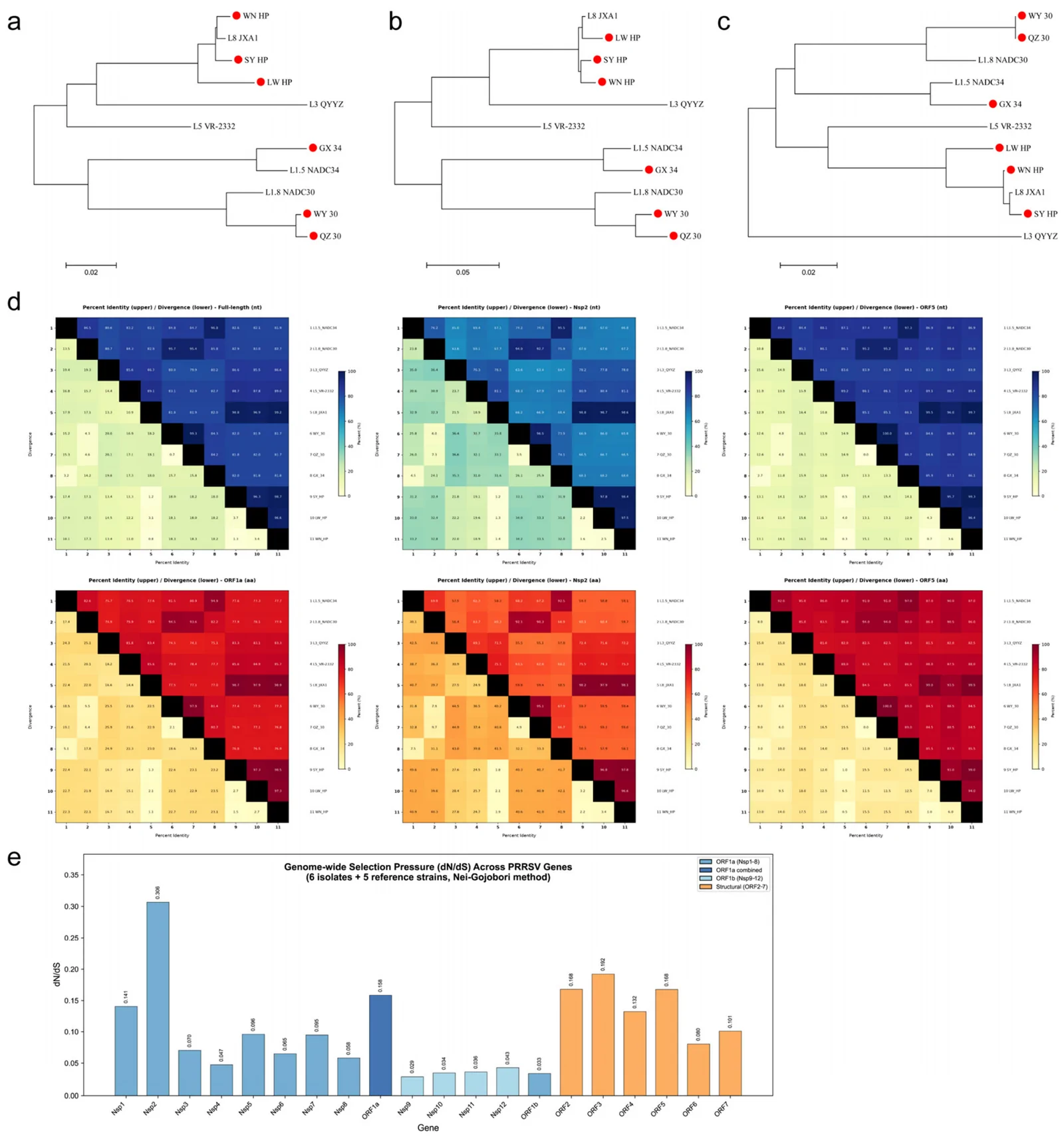
Genetic evolution analysis of six PRRSV isolates. (**a**–**c**) Maximum Likelihood trees based on complete genome (**a**), NSP2 (**b**), and ORF5 (**c**) nucleotide sequences. (**d**) Nucleotide and amino acid identities of the six isolates relative to reference strains. (**e**) Genome-wide dN/dS across 18 genes (six isolates and five reference strains; Nei–Gojobori method).

The six isolates spanned 15,013–15,347 nt (WY 30 and QZ 30: 15,013; GX 34: 15,112; SY HP: 15,320; LW HP and WN HP: 15,347) and exhibited the canonical PRRSV-2 genome organization (5′UTR–ORF1a/ORF1b–ORF2a/2b–ORF3–ORF4–ORF5–ORF6–ORF7–3′UTR) (Supplementary Table S5). Phylogenetic trees based on the complete genome, NSP2, and ORF5 consistently assigned WY 30 and QZ 30 to NADC30-like (sublineage 1.8), GX 34 to NADC34-like (sublineage 1.5), and SY HP, LW HP, and WN HP to HP-PRRSV-like (lineage 8) (Figure 4a–c). Pairwise comparisons revealed 82.1–89.6% full-length identity across different lineages, and 95.5–99.2% identity within the same lineage. WY 30 and QZ 30 shared 96% and 95% identity with NADC30, respectively; GX 34 shared 97% with NADC34; and SY HP, LW HP, and WN HP shared 99%, 97%, and 99% with JXA1, respectively. NSP2 was the least conserved gene (nt 64–99%; aa 55–98%), whereas NSP9 (RdRp) was the most conserved non-structural protein (aa 95–100%) (Figure 4d; Supplementary Table S6).

Gene-wise dN/dS ratios across 18 coding regions were all well below 1 (range: 0.0288–0.3065) indicating that purifying selection predominates across the genome. NSP2 had the highest ratio of any region analysed (dN/dS = 0.3065), identifying it as the least constrained of these regions and consistent with its role as a hotspot for deletions and inter-lineage divergence; this relative ranking is not, in itself, evidence of positive selection. Among non-structural proteins, NSP9 was the most constrained (dN/dS = 0.0288). ORF1b-encoded proteins (Nsp9–Nsp12) had the lowest mean dN/dS (0.0334), versus 0.1098 for ORF1a proteins (Nsp1–Nsp8) and 0.1402 for structural proteins (ORF2–ORF7). NSP2 accounted for most ORF1a variation, with its hypervariable region (aa 301–600) showing markedly elevated dN/dS relative to N- and C-terminal domains (Figure 4e; Supplementary Table S7).

### 3.5. GP5 Variation and Selection Pressure Analysis of PRRSV Isolates

**Figure 5:**
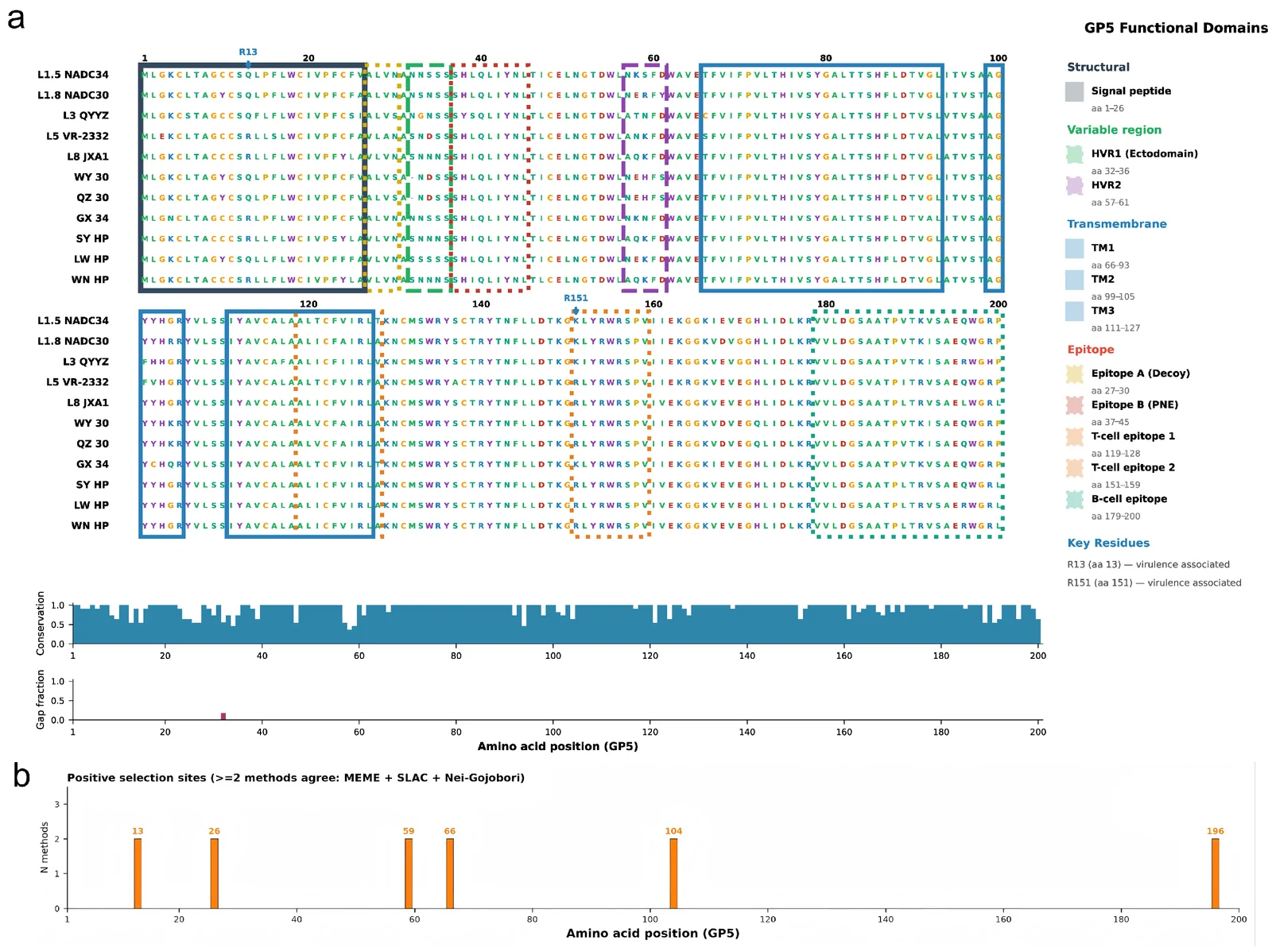
GP5 amino acid alignment and positively selected sites of six PRRSV isolates. (**a**) GP5 alignment, highlighting signal peptide, HVR1/HVR2, TM1–3, and antigenic epitopes. (**b**) Positively selected sites supported by ≥2 of 3 methods (MEME, SLAC, Nei–Gojobori).

GP5 alignment revealed marked variation in antigenic regions. At virulence-associated residues, R13Q occurred in lineage 1.8 strains, and R151K in lineage 1.5 strains. The signal peptide (aa 1–26) and TM1–TM3 were highly conserved, whereas HVR1 (aa 32–36) and HVR2 (aa 57–61) showed extensive substitutions; one NADC30-like isolate carried a deletion at position 32 within HVR1. The PNE (aa 37–45) and the predicted N-glycosylation sites N44 and N51 were largely conserved across all strains (Figure 5a). Selection pressure analysis of the six isolates plus five reference strains using MEME, SLAC, and Nei–Gojobori identified six sites supported by ≥2 methods (aa 13, 26, 59, 66, 104, and 196) (Figure 5b; Supplementary Table S8). Of these, aa 13 and aa 151 (the latter by Nei–Gojobori alone) correspond to previously reported virulence-associated residues, with R13Q and R151K prevalent in lineage 1. Site aa 59 in HVR2 was also identified in the 136-sequence population. The clustering of selected sites in the signal peptide and HVR1/HVR2 suggests immune pressure and receptor adaptation as major drivers of GP5 evolution.

### 3.6. NSP2 Variation and Selection Pressure Analysis of PRRSV Isolates

**Figure 6:**
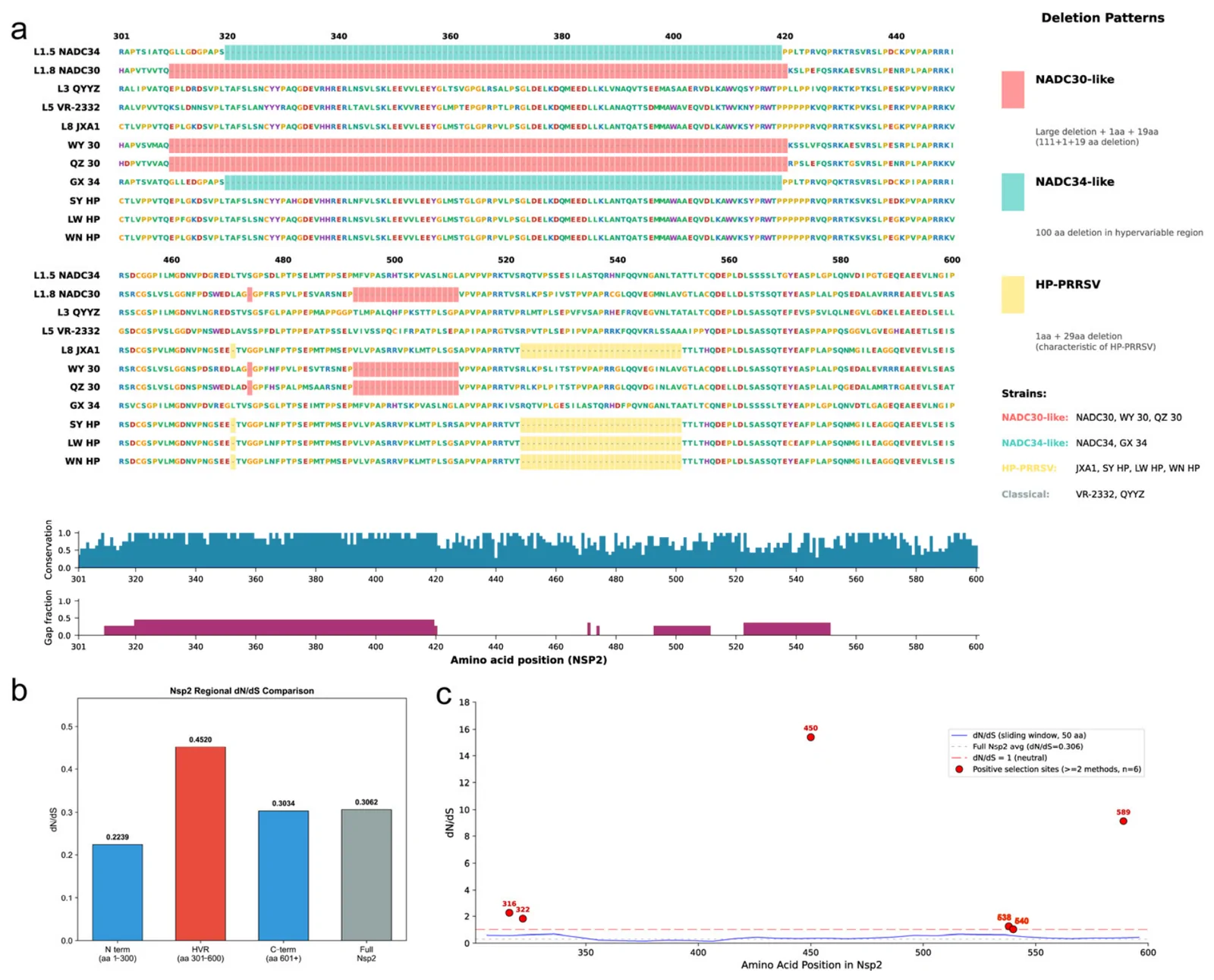
NSP2 hypervariable region variation and selection pressure. (**a**) Amino acid alignment of the NSP2 HVR (aa 301–600). (**b**) Sliding-window dN/dS profile across the HVR. (**c**) Positively selected sites identified by MEME and Nei–Gojobori.

NSP2 alignment of the six isolates against VR-2332 revealed lineage-specific deletion patterns in the hypervariable region (aa 301–600). GX 34 (NADC34-like) carried a characteristic 100-aa continuous deletion; WY 30 and QZ 30 (NADC30-like) harbored a 131-aa discontinuous deletion, a hallmark of sublineage 1.8; and SY HP, LW HP, and WN HP (HP-PRRSV-like) displayed the typical 30-aa deletion (Figure 6a). Sliding-window analysis showed that the HVR (aa 301–600) had the highest dN/dS (0.4520), though all regions remained below 1, consistent with predominant purifying selection and relaxed constraint specifically in the HVR (Figure 6b). MEME and Nei–Gojobori analyses identified 15 and 29 positively selected sites, respectively; six were concordant (aa 316, 322, 450, 538, 540, and 589) (Figure 6c; Supplementary Table S9). Of these, aa 450 showed the highest dN/dS (15.41), followed by aa 589 (9.13), indicating strong selection in the central and C-terminal HVR. Sites aa 316 and 322 localized within or adjacent to NADC30/NADC34-specific deletion regions, whereas aa 538 and 540 fell within the HP-PRRSV-specific deletion. The co-localization of selected sites with lineage-specific deletions suggests that deletion accumulation and positive selection act synergistically to shape NSP2 diversity, potentially contributing to immune evasion and lineage-specific pathogenicity.

### 3.7. Recombination Analysis of PRRSV Isolates

**Figure 7:**
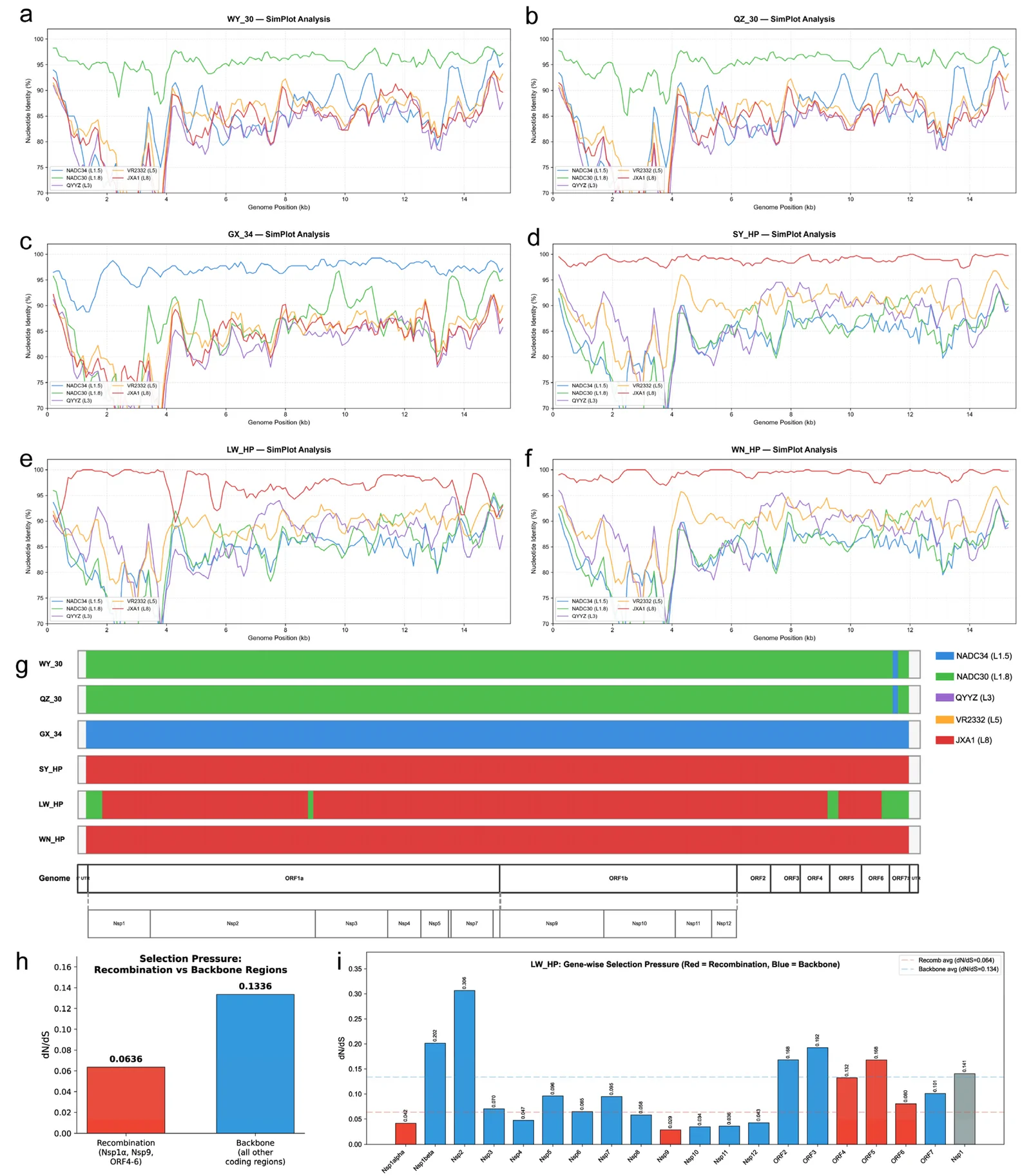
Recombination analysis of six PRRSV isolates. (**a**–**f**) Sliding-window similarity plots (SimPlot) of six isolates against reference strains; *y*-axis, nucleotide identity (%); *x*-axis, genome position (kb). (**g**) Schematic of genome architecture and putative recombination patterns. (**h**) dN/dS comparison between recombinant regions (Nsp1α, Nsp9, ORF4–6) and backbone regions of LW HP. (**i**) Gene-wise dN/dS of LW HP, with recombinant genes (red) showing lower dN/dS than backbone genes (blue). Recombination patterns are shown as similarity to the reference strains analysed and do not identify the parental viruses; BootScan plots are provided in Supplementary Figure S1.

Recombination analysis of the whole genomes of six isolates using RDP4 and SimPlot revealed limited evidence of recombination. GX 34 showed 97% nucleotide identity with the NADC34 reference strain across the genome. SY HP and WN HP were likewise closely related to JXA1 throughout the genome (99% identity) and showed no detectable recombination. WY 30 and QZ 30 were predominantly NADC30-like (95–96% genome-wide identity) but carried a short segment showing highest similarity to NADC34 at the extreme 3′ terminus (ORF7/3′UTR, ~nt 15,100–15,200). In LW HP, RDP4 and sliding-window analysis concordantly identified six breakpoints at ~nt 520, 4280, 4440, 13,880, 14,120, and 15,000 (Figure 7a–f; Supplementary Figure S1). Its genomic backbone showed the highest nucleotide identity to JXA1 (~97%), with short NADC30-like insertions at the 5′ Nsp1α region (~nt 190–520), within ORF1b/Nsp9 (~nt 4280–4440), and in the 3′ ORF4–ORF6 region (~nt 13,880–15,000) (Figure 7g). BootScan analysis with the same reference set independently supported these breakpoints, with bootstrap support for JXA1 falling and support for NADC30 rising at the corresponding coordinates (Supplementary Figure S1). Recombinant regions showed lower dN/dS (0.0636) than the backbone (0.1336), with gene-wise analysis confirming stronger purifying selection in recombinant genes; NSP2 retained the highest dN/dS (0.308) among all annotated genes (Figure 7h–i; Supplementary Table S10).

## 4. Discussion

PRRSV has circulated in China for nearly three decades, its epidemiological landscape continually reshaped by mutation and recombination. As a major swine-producing hub in central China, Hubei endures substantial PRRSV burden. The present study employed a tiered surveillance strategy: universal RT-PCR of the conserved ORF7/3′UTR for broad screening, ORF5 sequencing for lineage classification, and whole-genome sequencing of representative isolates for refined variation and recombination analysis. This design couples large-scale screening reliability with high-resolution characterization of circulating strains.

The overall positivity among the samples tested in this study was 26.32% (389/1478; 2024–2025). Published values for other Chinese regions differ substantially. For example ~40.6% in southern China (2022–2024) [51], 28.16% in Sichuan (2024) [52], 18.42% in northern China (2021–2023) [53] and 19.72% in Henan and Shanxi (2023–2024) [54]. But these figures are not directly interchangeable with ours. Even for the closest available comparison, which like this study analysed clinical samples from suspected-infected farms, the reported positivity was obtained with real-time RT-PCR, from a different specimen mix, and in different provinces and years; the other studies likewise differ in sampling frame, specimen type, herd clinical status and assay format, and some measure antibody rather than viral RNA. The value reported here is therefore a positivity rate among submitted clinical samples from pigs with clinical signs, describing the diagnostic yield of this surveillance stream rather than the prevalence of PRRSV in the Hubei pig population. Accordingly, we draw no inference about relative regional prevalence from these numbers; the observation that the value lies between those reported elsewhere simply indicates that PRRSV circulation in central China continues at a level warranting sustained surveillance.

Nursery and growing-finishing pigs accounted for 63.76% of positives, consistent with nationwide patterns [55], indicating that growing pigs represent the predominant population in which PRRSV is detected in this cross-sectional survey. The high detection rate in nursery pigs (32.65%) coincides with the weaning-to-mixing period, when persistent subclinical infection and horizontal spread are commonly observed. All-in/all-out production, consistent gilt acclimatization, and segregated early weaning warrant emphasis in Hubei herds. Detection rates differed among cities (range 0–60.00%; χ^2^ = 88.35, *p* < 0.001); however, this observation is subject to two constraints and is reported as hypothesis-generating. First, the number of samples per city was highly unequal (10–598) and sampling was clinically driven rather than random, so these values reflect submission and detection patterns and cannot be read as regional prevalence. Second, no farm-level biosecurity, management or pig-movement data were collected in this study, and we therefore do not attribute the observed heterogeneity to on-farm biosecurity or to any other specific determinant. Distinguishing between sampling artefacts and genuine geographic differences will require a dedicated survey with random or proportional sampling and the simultaneous collection of farm-level covariates.

The ORF7/3′UTR is highly conserved across PRRSV-2 lineages and is an established target for universal detection [56,57]. The primer pair used here spans this conserved junction, minimizing false negatives from hypervariable-region mutations and suiting large-scale surveillance in regions of mixed lineage composition. Compared with multi-primer differential assays, this single-pair format offers greater stability and lower cost for primary screening, with lineage assignment deferred to ORF5 sequencing. Phylogenetic analysis showed that lineage 1 strains (NADC30-like and NADC34-like) were the predominant lineage among the 136 ORF5 sequences obtained in this 2024–2025 surveillance period (66.18%), consistent with the nationwide lineage replacement documented recently [40,51,52,54]. A recent nationwide survey of 328 samples from 27 provinces (2024–2025) confirmed sublineage 1.8 as dominant, with Hubei emerging as a transmission cluster for both 1.8 and 1.5 [3]. Notably, sublineage 1.5 accounted for 27.94% in Hubei—above the national average of 23.2% [3], consistent with the circulation of NADC34-like PRRSV in central China during the study period. HP-PRRSV (lineage 8) persists at 24.26%, above the national average [36], signifying that classical highly pathogenic strains remain a substantive outbreak risk in this region.

Whole-genome analysis revealed lineage-specific NSP2 deletions: a 100-aa continuous deletion in NADC34-like strains, a 131-aa discontinuous deletion in NADC30-like strains, and a 30-aa deletion in HP-PRRSV strains [28,36,58]. These deletion patterns represent established lineage-specific molecular signatures and serve as reliable markers for differential diagnosis. The 30-aa NSP2 deletion is shared by the JXA1 field strain and the JXA1-R vaccine, because the vaccine was derived from this strain; it is therefore a lineage marker but does not distinguish field virulent from vaccine-derived lineage 8 viruses. Because JXA1-R differs from its parent JXA1 by only ~0.7% of the genome, percentage identity alone is likewise uninformative; we therefore compared the two non-recombinant lineage 8 isolates (SY HP and WN HP) with the vaccine at the positions that separate JXA1-R from independent lineage 8 field strains. Both carried the vaccine allele at only 1/57 and 6/57 of these positions, respectively (Supplementary Table S11), indicating that they are field-virulent lineage 8 strains rather than modified-live vaccine derivatives [53,59,60]. Contrary to reports of frequent inter-lineage recombination in the field [36,53], RDP4 and sliding-window analyses showed that GX 34, SY HP, and WN HP were genetically most similar to their prototype strains (NADC34 and JXA1), whereas WY 30 and QZ 30 carried a short NADC34-derived segment at the 3′ ORF7/3′UTR. Only LW HP showed substantial recombination: a JXA1-like backbone interspersed with short NADC30-like fragments at the 5′ terminus, Nsp9, and ORF4–ORF6. The low recombination rate likely reflects limited sampling and the stochasticity of co-infection rather than an absence of field recombination. Notably, LW HP indicates that viruses of different lineages have co-infected hosts at some point in the ancestry of this strain, consistent with the co-circulation of five lineages in Hubei. The lower dN/dS in the recombinant segments of LW HP (0.0636) compared with its backbone (0.1336) is consistent with the possibility that chimeric genomes may be subject to heightened functional constraints. However, because this observation is based on a single recombinant isolate, it should be regarded as a hypothesis rather than a demonstrated mechanism. The scarcity of detectable inter-lineage recombinants in the present dataset may reflect multiple non-exclusive factors, including limited sampling, the stochasticity of co-infection events, and the possibility that many recombinant progeny are non-viable or attenuated. Larger genomic datasets are required to test these possibilities. Nevertheless, co-circulation of five lineages (1.8, 1.5, 8, 5, and 3) provides ample opportunity for genetic exchange, and ORF1a and ORF2–ORF3 are known recombination hotspots [3]. Continuous whole-genome surveillance is thus warranted for early detection of emergent recombinants with altered virulence or immune-escape potential.

Selection pressure analysis further delineated the evolutionary dynamics of circulating strains. Genome-wide dN/dS ratios were highest for NSP2 (0.3065), consistent with its role as the primary hotspot for lineage-specific deletions and immune evasion [28,36]. Because all ratios remained well below 1, this ranking indicates reduced constraint rather than detectable gene-level positive selection; evidence of positive selection in our data is confined to individual codons in GP5 and the NSP2 HVR. By contrast, NSP9 (RdRp) was the most conserved (0.0288), reflecting strong functional constraint on the replication complex. In GP5, aa 32 and aa 59 were concordantly identified by all five methods (FEL, FUBAR, MEME, SLAC, Nei–Gojobori), localizing to HVR1 and HVR2, respectively, both proximal to known antigenic epitopes [33,36]. The clustering of positively selected sites in HVR1 and HVR2 is consistent with the hypothesis that host immune pressure contributes to GP5 sequence diversification. However, the present study is based on sequence analysis alone and does not include serological neutralization or cross-protection experiments; therefore, we cannot directly conclude that these substitutions alter vaccine-induced protection. The observed amino acid divergence in the decoy epitope, PNE, and HVRs, together with positively selected residues at aa 32 and aa 59, warrants further investigation through virus-neutralization assays and cross-protection studies to assess whether vaccine-induced immunity is being eroded by the evolving field strains. Continuous antigenic matching between circulating strains and vaccine candidates should therefore be prioritized in regional immunization programs.

In the NSP2 HVR (aa 301–600), six sites (aa 316, 322, 450, 538, 540, and 589) were concordantly detected by MEME and Nei–Gojobori, with aa 450 yielding the highest dN/dS (15.41). Notably, several selected sites overlapped lineage-specific deletion regions, suggesting that deletions and positive selection act synergistically to shape NSP2 diversity and immune evasion. Intriguingly, LW HP showed lower dN/dS in recombinant segments (0.0636) than in the backbone (0.1336), implying that acquired NADC30-like fragments are under heightened purifying selection, possibly reflecting functional constraints on protein–protein interactions in the chimeric genome. Given that a single recombinant was analysed, this interpretation should be regarded as tentative.

Six strains were isolated on primary PAMs and adapted to MARC-145 cells, including NADC30-like (WY 30, QZ 30) and NADC34-like (GX 34) variants generally regarded as fastidious in immortalized lines [61,62]. This cross-adaptation may reflect the relatively recent emergence of lineage 1 in Hubei, where ongoing circulation has favored phenotypes amenable to laboratory culture. TEM confirmed the isolates at the ultrastructural level: 50–60 nm enveloped spherical particles within ER-derived vesicles and abundant DMVs, consistent with canonical PRRSV morphology [62]. Mitochondrial swelling and ER dilation reflect the cytopathology and cellular stress induced by PRRSV replication, providing morphological groundwork for future studies of pathogenesis and virus–host interactions.

Five passages in cell culture (two on PAMs, three on MARC-145) may introduce minor adaptive mutations. However, several considerations mitigate this concern. First, the lineage-defining NSP2 deletion patterns (100-aa in GX 34; 131-aa in WY 30/QZ 30; 30-aa in SY HP/LW HP/WN HP) are fully consistent with recent field-isolate reports [28,36,58], indicating that major genomic features were retained. Second, gene-wise dN/dS was calculated against five prototype reference strains; cell-adaptive mutations, if any, would not alter the relative ranking of evolutionary rates among genes. Third, the 136 ORF5 sequences for selection pressure analysis were derived directly from clinical specimens, and the key positively selected sites (GP5 aa 32 and aa 59) were concordantly identified by all five methods. Nevertheless, minor strain-specific mutations during P1–P5 cannot be excluded, and future studies should sequence directly from clinical specimens to confirm the absence of cell-culture-adaptive changes.

The representativeness of the whole-genome data warrants comment. The six genomes subjected to whole-genome sequencing represent three of the five lineages detected by ORF5 sequencing (sublineages 1.8/L1C, 1.5/L1A and lineage 8.7/L8E), which together account for 90.44% (123/136) of the field sequences; lineage 5/L5 (5.15%) and lineage 3/L3 (4.41%) were not recovered as complete genomes. The lineages represented therefore correspond to those that were most readily recovered as complete genomes, rather than to a predefined lineage-balanced design, and the genomes should be regarded as representative of the dominant lineages rather than of the full genetic diversity of the ORF5 dataset. The ORF5 dataset is itself spatially unbalanced, and the whole-genome subset is likewise not a proportionally sampled geographic subset. Consequently, the recombination and selection-pressure estimates derived from these genomes describe the lineages and farms represented here and should not be extrapolated to the province as a whole; the low recombination rate observed is more plausibly explained by the limited number of genomes examined and by the stochasticity of co-infection than by an absence of field recombination.

Several limitations merit note. Although 12 prefecture-level cities were sampled, mountainous regions yielded fewer specimens, potentially constraining the representativeness of province-wide estimates. City-level detection rates were derived from clinically submitted samples using a non-probabilistic design with markedly unequal sample numbers (10–598 per city); they should not be interpreted as regional prevalence, and between-city differences cannot be attributed to specific management or biosecurity factors on the basis of these data. ORF5 was sequenced unidirectionally, and only six isolates were fully sequenced. These six isolates cover three of the five lineages detected, accounting for 90.44% of the ORF5 sequences, but they do not include lineage 5/L5 or lineage 3/L3. In addition, the whole-genome subset is not geographically proportional to the ORF5 dataset. Additional complete genomes, particularly from the minority lineages and from under-sampled cities, are required to resolve recombination hotspots and evolutionary dynamics at higher resolution. The pathogenicity of these isolates in piglets also awaits in vivo evaluation. Future work should expand sampling, conduct systematic pathogenicity comparisons across lineages, and assess cross-protection of commercial vaccines against local strains.

## 5. Conclusions

This study delineates the detection rates and genetic characteristics of PRRSV in Hubei during 2024–2025. Among the samples examined during 2024–2025, lineage 1 strains (NADC30-like and NADC34-like) were predominant (66.18%), whereas HP-PRRSV persisted at 24.26%. Whole-genome analysis revealed lineage-specific NSP2 deletions and generally limited recombination, with LW HP showing a JXA1-like backbone bearing NADC30-like insertions. Selection pressure analysis identified NSP2 as the least constrained coding region, with positively selected sites in GP5 and the NSP2 HVR. Six clinical isolates were recovered from field specimens, adapted to MARC-145 cells, and characterized ultrastructurally. These findings augment molecular epidemiological data for central China and provide critical strain resources for regional prevention, control, and vaccine development.

## Figures and Tables

**Table 1:** Number of clinical samples tested and PRRSV-positive samples, detection rate with 95% confidence interval, and share of the total sample set, by prefecture-level city, Hubei Province, 2024–2025.

City (Prefecture)	Samples Tested (*n*)	Positive Samples	Detection Rate (%)	95% CI (%)	Share of All Samples (%)
Huanggang	598	139	23.24	20.0–26.8	40.46
Wuhan	362	108	29.83	25.4–34.7	24.49
Xiangyang	189	51	26.98	21.2–33.7	12.79
Enshi	89	36	40.45	30.9–50.8	6.02
Xiaogan	55	0	0.00	0–6.5	3.72
Tianmen	42	12	28.57	17.2–43.6	2.84
Xianning	30	15	50.00	33.2–66.8	2.03
Yichang	30	16	53.33	36.1–69.8	2.03
Huangshi	30	0	0.00	0–11.4	2.03
Jingmen	23	0	0.00	0–14.3	1.56
Qianjiang	20	12	60.00	38.7–78.1	1.35
Shiyan	10	0	0.00	0–27.8	0.68
Total	1478	389	26.32	24.1–28.6	100

Note. Sampling was clinically driven (samples submitted for diagnostic testing) rather than randomly or proportionally allocated; the rates are therefore detection rates among submitted samples and not estimates of regional prevalence.

## Data Availability

The genome sequences generated in this study, along with all supporting analytical data (sequence alignments, selection pressure results, and identity matrices), are provided in the Supplementary Materials. All relevant data are available from the corresponding author upon reasonable request.

## Supplementary Materials

The following supporting information can be downloaded at: https://www.mdpi.com/article/10.3390/ani16213367/s1. Supplementary Table S1: PRRSV sample information and detection results in Hubei Province. Supplementary Table S2: ORF5 nucleotide and amino acid identity matrices. Supplementary Table S3: Positively selected sites in GP5. Supplementary Table S4: GP5 domain-wise amino acid variation rates across lineages. Supplementary Table S5: Genome organization and gene sequences of six PRRSV isolates. Supplementary Table S6: Nucleotide and amino acid identities of isolates across genomic regions relative to reference strains. Supplementary Table S7: Gene-wise dN/dS ratios across the PRRSV genome. Supplementary Table S8: Positively selected sites in GP5 of isolates. Supplementary Table S9: Positively selected sites in the NSP2 hypervariable region (aa 301–600) of isolates. Supplementary Table S10: dN/dS comparison between recombinant and backbone regions of LW HP. Supplementary Table S11: Comparison of the six isolates with JXA1-R and other vaccine strains. Supplementary Figure S1: BootScan analysis of the six PRRSV isolates.

## Abbreviations

The following abbreviations are used in this manuscript:

aa	amino acid
CPE	cytopathic effect
DMEM	Dulbecco’s modified Eagle’s medium
DMV	double-membrane vesicle
dN/dS	ratio of non-synonymous to synonymous substitution rates
FBS	fetal bovine serum
FEL	Fixed Effects Likelihood
FUBAR	Fast Unconstrained Bayesian Approximation
GP5	glycoprotein 5
HP-PRRSV	highly pathogenic PRRSV
HVR	hypervariable region
HyPhy	Hypothesis Testing Using Phylogenies
IFA	indirect immunofluorescence assay
InDel	insertion-deletion
MEME	Mixed Effects Model of Evolution
MOI	multiplicity of infection
ML	Maximum Likelihood
Nsp	non-structural protein
nt	nucleotide
ORF	open reading frame
PAM	porcine alveolar macrophage
PBS	phosphate-buffered saline
PNE	primary neutralizing epitope
PRRS	porcine reproductive and respiratory syndrome
PRRSV	porcine reproductive and respiratory syndrome virus
RdRp	RNA-dependent RNA polymerase
RDP	Recombination Detection Program
RT-PCR	reverse transcription-polymerase chain reaction
SLAC	Single-Likelihood Ancestor Counting
SNP	single nucleotide polymorphism
TCID_50_	50% tissue culture infectious dose
TEM	transmission electron microscopy
TM	transmembrane domain
UTR	untranslated region
